# Elevational biodiversity gradients in the Neotropics: Perspectives from freshwater caddisflies (Insecta: Trichoptera)

**DOI:** 10.1371/journal.pone.0272229

**Published:** 2022-08-05

**Authors:** Blanca Ríos-Touma, Francisco Cuesta, Ernesto Rázuri-Gonzales, Ralph Holzenthal, Andrea Tapia, Marco Calderón-Loor

**Affiliations:** 1 Facultad de Ingenierías y Ciencias Aplicadas, Ingeniería Ambiental/ Grupo de Investigación en Biodiversidad, Medio Ambiente y Salud–BIOMAS-, Universidad de Las Américas, Quito, Ecuador; 2 Department of Entomology, University of Minnesota, St. Paul, Minnesota, United States of America; 3 Department of Terrestrial Zoology, Entomology III, Senckenberg Research Institute and Natural History Museum, Frankfurt, Germany; 4 Mashpi Reserve, Quito, Ecuador; 5 Centre for Integrative Ecology, School of Life and Environmental Sciences, Deakin University, Melbourne, Australia; University of Eldoret, KENYA

## Abstract

Aquatic insects in the order Trichoptera are extremely diverse in number of species and their trophic roles. However, their distribution and diversity patterns are poorly known in the Neotropics, including the species restricted to tropical mountain ecosystems. Recent studies in tropical mountains have shown high levels of endemism of aquatic insects and changes in the composition of communities over short distances. Still, the incidence of environmental filters that explain such patterns has not been addressed quantitatively. Given the relevance of understanding Trichoptera spatial diversity patterns to prioritize conservation areas for freshwaters, as well as to obtain baseline information to predict changes in aquatic communities facing global environmental changes, we assessed the species distribution and assemblages of caddisflies along an elevational gradient from 600 to 3,600 m a.s.l. on the equatorial Andes. In this area, we had long-term continuous climate data with hourly resolution. We collected adult caddisflies in seven localities along this gradient using light traps. We sampled each locality for two hours after sunset for three consecutive days. All specimens collected were identified to species or morphospecies. Our results showed an increase in species and genera numbers with decreasing altitude, albeit no significant. Minimum air temperature is the main environmental variable explaining Trichoptera community assemblages. β‐diversity (taxon turnover among sites), as opposed to species richness, increased with altitude and showed a bimodal distribution along the elevation gradient for both genera and species assemblages, which resulted in a significant shift in community composition of species and genera at 2,000 m a.s.l. Our null-models confirm the observed patterns of B-diversity are non-random and suggest a strong environmental filtering of tropical caddisflies community assemblies and turnover. Geographic distance coupled with changes in environmental conditions along the elevation gradient explained a high percentage of community variance, as documented for other taxa (e.g., vascular plants), suggesting the importance of securing habitat connectivity along the altitudinal gradient to protect aquatic insect diversity effectively.

## Introduction

Andean mountains provide an opportunity to examine the relationship between diversity patterns and community assemblages with environmental filters, such as elevation, topography, and temperature [1–3]. Previous studies of these patterns have found distinctive spatial organization depending on the taxa under study. Generally, plant and animal species richness show a hump-shaped pattern, with maxima at intermediate elevations between 500–2,000 m a.s.l. [1, 4–6]. Additionally, a monotonic decrease has also been observed [6–8]. Conversely, mid-elevation diversity peaks can occur when the elevation gradient is truncated on part of the elevational gradient [9]. For birds, local species richness in a mountain forest in Bolivia peaked at around 1,000 m a.s.l., declined sharply near 1,750 m a.s.l., and then remained relatively constant [1]. In this study, elevation was the best single predictor, accounting for 78–85% of the variance in the data set. Herzog et al. [1] reported that a richness peak at 1,000 m a.s.l. resulted from an overlap of two distinct avifaunas (lowland and highland). Also, a recent study of fern diversity patterns across the tropics found that elevational richness patterns were symmetrically hump-shaped and overall richness was virtually equal along most of the mountains scattered along the latitudinal gradient [5].

The number of narrow-range species (i.e., endemics) along elevational gradients has also been examined. However, this has been done to a much lesser extent. For example, Kessler [10] focused on the patterns of plant endemism between different taxa along an Andean elevation gradient. In that study, he reported that most groups had hump-shaped patterns with maxima at different elevations and mainly at the same or higher elevations than species richness maxima. Most taxa had the highest concentration of endemics in the narrowest and most fragmented elevational belts (> 2,000 m a.s.l.), presumably because of the fragmentation of species populations, the increase in niche partitioning, and harsher environmental conditions. Carvajal-Quintero et al. [11] found a similar pattern with a high concentration of endemic fish species at higher elevation due to the distance between headwater streams. Fjeldså et al. [12], in their analyses of bird endemism patterns across the Andes, documented that distribution patterns of endemic species along tropical elevational gradients are different from those of species richness and usually peak above 2,000 m a.s.l. The spatial patterns of endemism found in this study were inverse to species richness patterns and were presumably controlled by area, topography, ecoclimatic stability, and taxon-specific ecological traits. In a recent study of Andean aquatic insect clades, Polato et al. [13] found that insect species from lower elevations had markedly narrower thermal tolerances and lower dispersal capacity than high altitude species. These factors result in significantly greater population divergence (allopatric speciation), higher cryptic diversity, higher speciation rates, and greater accumulation of species over time in lower elevations than in mid and high elevations.

The patterns observed in birds, mammals, and plants have been studied scarcely in freshwater organisms in this region [11, 14, 15]. Although longitudinal connectivity among river networks exists, elevation and other environmental gradients such as temperature and precipitation could act as barriers for freshwater species. Additionally, there is a lack of knowledge on the taxonomy and life history of freshwater insects, which is essential for understanding these patterns. For example, Holzenthal et al. [16] estimated that only about 30% of Trichoptera species in the tropical Andes are known to science.

Caddisflies occupy a great variety of habitats, have diverse trophic preferences, and are very sensitive to environmental changes [17]. In Ecuador, 314 species are currently recorded, but estimations predict that this may only represent about 50% of the true diversity. From these species records, the majority of them occur below 2000 m asl [18]. In an era of climate and land-use change, it is essential to know the actual diversity, distribution, and life history of caddisflies in the Neotropics due to their sensitivity to environmental changes [19]. In this context, our goal was to assess which effect environmental variables have in shaping Trichoptera species and genera distribution and community assemblages along an elevation gradient in the equatorial Andes. We expected: (1) a decrease in species and generic diversity with increasing elevation, and (2) an increase in caddisfly community composition dissimilarity as the elevation and geographic distance increase. Thus, our objectives are: (1) to determine the variation in the number of species and genera of Trichoptera along the elevational gradient starting at the foothills of the Pichincha volcano (3,500 m a.s.l.) and following the Guayllabamba watershed down to 600 m a.s.l., (2) to evaluate the role of environmental variables in Trichoptera community composition, and (3) to assess the importance of geographic proximity and elevation in explaining the composition of caddisflies assemblages and community composition turnover (β‐diversity) patterns along these gradients.

## Materials and methods

### Study area

The study area is in the remnant montane cloud forest on the western flank of the Pichincha volcano in the Ecuadorian Andes. This study took advantage of a long‐term forest biodiversity and carbon monitoring transect established in 2015 by Pinto & Cuesta [20]. The permanent transect spans 3,000 m of elevation, from the piedmont forest (619 m a.s.l.) to upper montane forest (3,464 m a.s.l.). The transect captures four ecosystem types (Piedmont Forest (PMF), Lower Montane Forest (LMF), Montane Forest (MF), and Upper Montane Forest (UMF) [21] that reflect changes in environmental conditions related to environmental gradients in mean annual temperature (7.2–21.6°C), relative air humidity (96.1%– 99.8%), and mean annual precipitation (1,580–2,448 mm; [22, 23])

**Fig 1:**
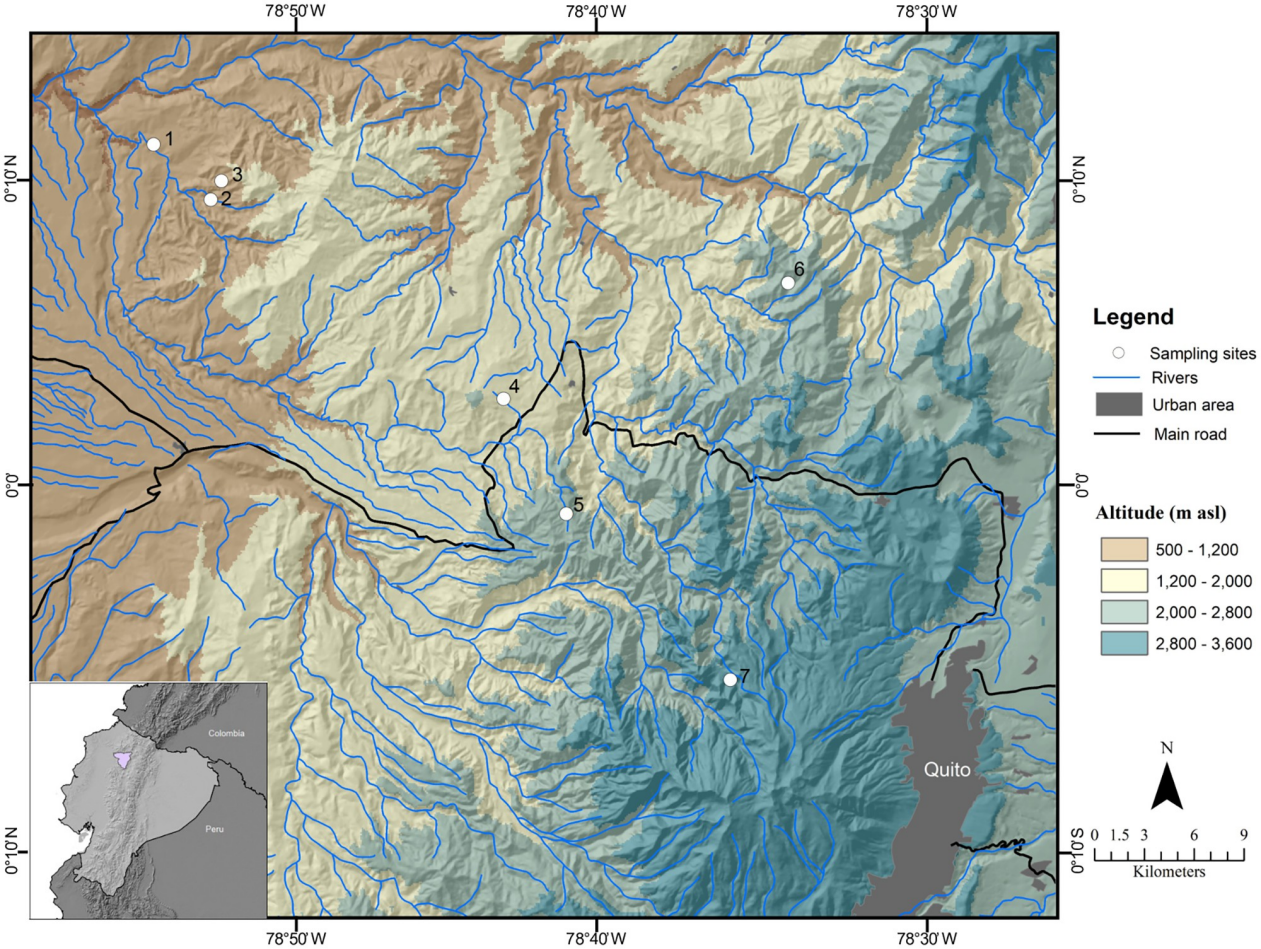
Map of the study localities in the Guayllabamba watershed across an elevation gradient (600–3,500 m a.s.l.) in the equatorial Andes. 1) Mashpi-Shungo reserve; 2) Mashpi Lodge locality 1; 3) Mashpi Lodge locality T; 4) Intillacta Reserve; 5) Bellavista Reserve; 6) Cedral Reserve; 7) Verdecocha Reserve. Map based on based on a freely available Digital Elevation Model produced by the Shuttle Radar Topography Mission (SRTM)–NASA: https://www2.jpl.nasa.gov/srtm/ext.

We selected 7 locations along the monitoring transect associated with second-order streams (Fig 1, Table 1). Locations were selected based on the following criteria: (1) a second-order stream was located at less than 1 km from the permanent forest plot, (2) the selected locations were distributed approximately every 600 meters, allowing to capture the variations of the microenvironmental conditions found along the elevation gradient (see [22])., (3) the riparian ecosystem was in good condition and did not show human impacts (S1 Fig). Although the Intillacta location has a considerable amount of agricultural land in its drainage area (15%), the riparian forest is well preserved and the stream water quality is good. Moreover, the Intillacta location is embedded in a private reserve that protects a montane forest fragment of 250 hectares.

**Table 1:** Study localities in the Guayllabamba watershed across an elevation gradient (600–3,500 m a.s.l.) in the equatorial Andes and environmental parameters. T = temperature.

Locality	Mean	Elevation	Natural	Agricultural	Drainage	Slope	Mean T	Average
	Precipitation	(m)	Vegetation	Cover	Area	(°)	°C	Minimum
	(mm)		(%)*	(%)*	(ha)			T °C
Mashpishungo	1595	619	94	6	15.21	14.0	21.64	20.25
Mashpi 1	2176	903	100	0	27.36	33.5	19.43	19.32
Mashpi T	2400	1028	100	0	339	31.8	20.6	19.42
Intillacta	1983	1874	85	15	68.1	18.9	15.98	14
Bellavista	1405	2281	100	0	36.3	24.8	13.61	11.92
Cedral	1247	2325	100	0	63	24.3	13	12.81
Verdecocha	1089	3464	99	1	554.04	35.0	9.8	8.1

*calculated for drainage area

### Collecting methods

At each locality, adult caddisflies were attracted to black (UV) fluorescent lights (F8T5/BL, 8 watts) placed across white plastic pans filled with 96% ethanol as a preserving fluid [19]. One pan trap with a black light was placed on the riverbank per locality each night. Sampling was conducted from 6 to 8 pm during three consecutive nights, from late June to early August 2017 (dry season; S1 Table). The dates were as far away from the full moon as possible to maximize the effectiveness of the traps [19]. All collected material was preserved in 96% ethanol until sorting and identification.

The material from each locality and date was initially sorted by “morphospecies” based on coloration, size, and habitus of the individuals. Additional characters such as the presence or absence of ocelli, the structure of maxillary palpi, the number of leg spurs, and the size and shape of antennae were used to further refine the sorting process [23]. Within each series, specimens were sorted by sex, and the abdomen of a few males per series were removed and cleared using the lactic acid method proposed by Blahnik et al. [24]. These male genitalia were then compared to the original species descriptions to identify them to the most precise taxonomic category possible. If identification to species level was not possible, taxa were considered as morphospecies within each genus [23].

To assess the influence of habitat variables, we constructed an environmental matrix used for the non-metric multidimensional scaling analysis. Relative humidity (RH), Mean (Mean T°) and minimum average annual air temperature (Min T°) were derived from HOBO Pro Temp/RH data loggers (U23-001; Onset, USA) in one-hour intervals at 1 m above ground level collected at each location between 2016–2018 [22]. Average annual precipitation (hereafter “precipitation”) was obtained from the Climatologies at high resolution for the Earth’s land surface areas (CHELSA) dataset at 30 Arc-sec resolution (~1km) [25]. Elevation, slope, and drainage area of each location were derived from the Shuttle Radar Topography Mission (SRTM) at 30 m pixel resolution, and land cover of each location was derived from pre-existing vegetation maps for the year 2016 derived from Landsat images at 30 m pixel resolution [26]

### Data analysis

#### Species diversity

We estimated species richness and species diversity (α-diversity) based on the Shannon-Weiner exponential (N1, Hill number 1) [28]. To assess sampling completeness (i.e., the number of species collected per sampling location), we used species and genera accumulation plots with the Chao 1 estimator and sample completeness estimator to assess the completeness of the sampling at each locality (elevation), which allows better comparison among assemblages [27–29]. This calculations were obtained using iNEXT online software [30]. To complement these sampling completeness calculations, we performed species accumulations curves with the observed species and Chao 1 estimator at the three samples at each site permuted, using PRIMER 6 software [31].

#### Community composition analysis

We analyzed patterns of community composition (species and genus abundance patterns) along the altitudinal gradient, for which we constructed an abundance matrix for each Trichoptera species and locality. We square-root transformed taxa abundances to increase the weight of low-abundance taxa in the analysis (68% of the species were represented by 2 or fewer individuals). We then constructed a between-locality Bray-Curtis similarity matrix as a similarity measure and performed a non-metric multidimensional scaling analysis (NMDS). Further, we performed a hierarchical agglomerative cluster analysis using a group average linkage procedure [31], and we assessed the significance of these groups using the Similarity Profile routine, SIMPROF, that tests data against the null hypothesis of absence of structure [32]. With the formed groups, according to the SIMPROF routine, we performed a SIMPER procedure to assess the dissimilarity among these groups and the genera and species responsible for the differences among groups [33]. These results were corroborated, correlating the ordination matrix with the genera/species abundance matrix (only Spearman correlations higher than 0.6 were included).

Lastly, we correlated the ordination matrix with environmental variables using Spearman correlations to address which variables were responsible for faunal groups (only correlations higher than 0.6 were included, and collinear environmental variables were discarded before correlations). All these analyses were performed using PRIMER 6 [31].

#### Geographic distance and β‐diversity

We also analyzed the importance of geographic distance in explaining community composition similarity between localities. For this, species and genera abundance matrices for each locality was built based on the sampling carried out at each locations (see sampling procedure). Then, we calculated a Bray-Curtis similarity matrix for species and genera between localities, using the same square-root transformed values described above, which were correlated with a geographic distance matrix using Spearman’s correlation coefficients in the RELATE procedure in PRIMER 6. We evaluated the significance of these correlations using a permutation procedure (9999 permutations). We chose this analysis over a classical Mantel test; in spatial analysis, the assumptions of linearity and homoscedasticity of the Mantel test do not hold in most cases [34, 35]. Further, the partial Mantel test interpoint distance values are treated independently as a single vector. Instead, the RELATE procedure works on the distance/dissimilarity matrix directly, and therefore it retains the spatial structure from the response data [34]. In addition, Trichoptera community turnover (i.e., β‐diversity) among the location along the elevation gradient was quantified for both species and genera, using the Whittaker index [36] as follows:

bw=S/∝−1

where, S is the total number of species recorded at both localities (S = *a* + *b* + *c*); component *a* comprises the number of shared species between both localities, and components *b* and *c* comprise the number of species present exclusively in each of the two localities; α is the mean number of species found within localities, expressed as (2a+b+c)/2. The index was calculated in R using the *vegan* package [37].

*Null models & Beta diversity*. To separate the contribution of local community assembly mechanisms from sampling effects owing to variation in species pools, we compared observed Whitaker β-diversity to values expected under a null model across the elevation gradient, and across the 3 elevation groups found in the NMDS and Cluster analysis [38–40]. Our null model accounts for regional sampling effects due to the size and structure of species pools. We used the algorithm *c0_both* implemented in the *commsim* function of the R *vegan* package, version 2.5–7 [37]. The *c0_both* model preserves the local richness and the species present at each locality, but individuals among species at each location are shuffled. Further, as the null model maintains the observed distributional patterns of species and their abundances, the observed dispersal limitation of species is conserved in the null model. We implemented 1,000 iterations of the null model, producing a frequency distribution of null β-diversity values for each locality across the elevation gradient. Based on this frequency distribution, we calculated a β-deviation or the standardized effect size (SES), using the mean and standard deviation of the null distribution as follows:

$$SES=\frac{X_{obs}-X_{null}}{s.\;d.\;\left(X_{null}\right)}X_{obs}$$

where X_obs_ is the observed dissimilarity value (i.e., Whitaker) between two communities, X_null_ the mean of the null distribution, and s.d. (X_null_) the standard deviation of the null distribution. SES is a standardized measure of the difference between observed and null β-diversity. It can be interpreted as the relative effect of local assembly mechanisms on β-diversity (e.g., environmental filtering) after removing the sampling effects from the observed species pools. Values greater than 1.96 (P < 0.05) indicate a higher-than-expected beta dissimilarity between the communities (i.e., non-random) and values below -1.96 indicate a lower than expected dissimilarity between the two communities (i.e., random). Higher positive SES values could be generated by functionally non-random replacement of species along the elevation gradient [40, 41].

## Results

### Diversity

We collected 595 individuals representing 70 species (including morphospecies) in 26 generic-level taxa (i.e., genera and subgenera) across the elevation gradient (S1 Table). The Chao 1 estimator and the sample coverage estimator (Table 2) suggest that samples from high elevation localities (> 2,325 m a.s.l.) are less complete (sample coverage between 0.65–0.72) compared to mid and lower elevation localities (with sample coverage between 0.79–0.96). These results indicate that our 3-day sampling strategy performed well at capturing local diversity in general. However, three localities collected less than 50% of the estimated richness, which agrees with the species accumulation curves, the low estimated richness collected coincides with no stabilization of species accumulation curves (S2 Fig). However, our sample coverage suggests that species richness is covered in a relatively good proportion, between 0.65 to 0.95 (S3 Fig).

**Table 2:** Biodiversity metrics across study localities.

Locality	Elevation (m)	Species	Genera	N	N1	Chao 1 (species)	Chao 1 (genera)	Sample Coverage
Mashpi- shungo	619	15	11	86	6.3	21	11	0.9424
Mashpi 1	903	7	5	24	3.5	7	7	0.7917
Mashpi T	1028	35	22	352	12.0	51	38	0.9575
Intillacta	1874	11	9	60	4.9	24	14	0.9172
Bellavista	2281	12	8	42	8.4	15	8	0.9081
Cedral	2325	9	6	17	6.9	27	11	0.6543
Verdecocha	3464	7	4	14	5.6	15	4	0.7249

N = abundance; N1 = Shannon-Wiener diversity index expressed as equally abundant species; Chao 1 = Chao 1 species/genera richness estimator; Sample Coverage = Estimated Sample Coverage, according to Chao and Jost 2012 [27].

### Genera across the elevation gradient

**Fig 2:**
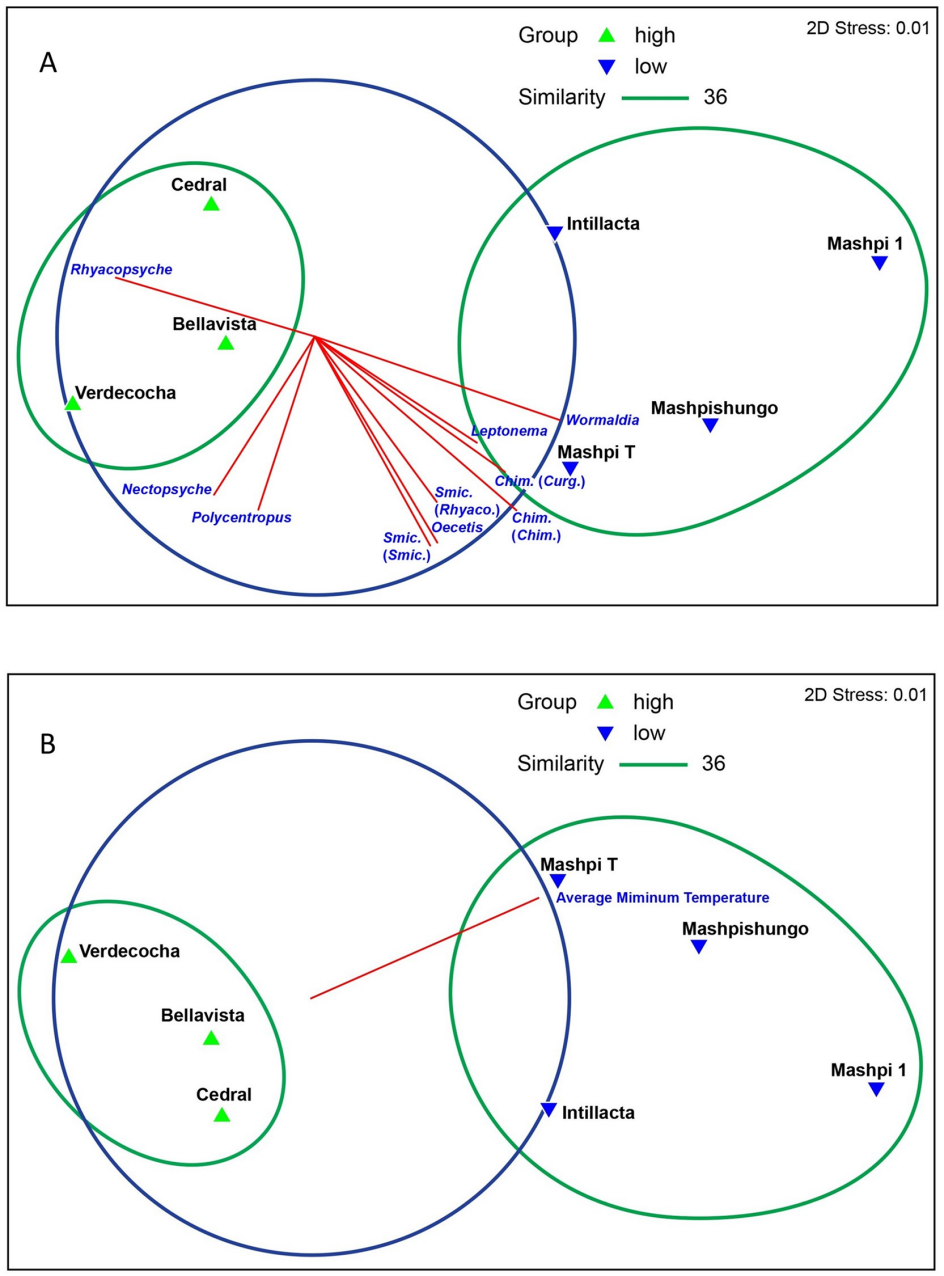
**A. Non-metric multidimensional scaling (NMDS) of Trichoptera genera assemblages across an elevation gradient (600–3,500 m a.s.l.) in western Ecuador**. Vectors represent the Spearman correlation (> 0.65) of the genera (with abundances square root transformed) with NMDS axis. **B. Non-metric multidimensional scaling of Trichoptera genera with correlations to environmental variables (> 0.65).**

At the generic level, two clusters were formed; one for genera below 2,000 m a.s.l. and the other for genera above this elevation (SIMPROF test, 5% significance level; Fig 2a, Table 3). These two clusters showed an average dissimilarity of 82%, according to the SIMPER analysis. The genera *Nectopsyche*, *Atopsyche*, *Helicopsyche*, *Amphoropsyche*, and *Rhyacopsyche* contributed the most to this dissimilarity in the high-elevation cluster, whereas *Leptonema*, *Chimarra*, *Wormaldia*, and *Smicridea* influenced the dissimilarity found in the low-elevation cluster (Fig 2a; Table 3). Nine genera/species were represented by single specimens from a single locality (S2 Table).

**Table 3:** SIMPER analysis between generic cluster groups (high and low altitude clusters) at localities across an elevation gradient (600–3,500 m a.s.l.), Volcán Pichincha, western Ecuador.

Genus	High	Low	Average	Distance/S	% Contribution	Cumulative %
	(average abundance)	(average abundance)	Distance			
*Leptonema*	0	4.86	13.24	2.81	16.16	16.16
*Chimarra*						
(*Chimarra*)	0	4.27	11.17	2.09	13.63	29.79
*Wormaldia*	0	2.69	9.4	11.34	11.47	41.26
*Nectopsyche*	3.14	1.62	7.18	1.05	8.76	50.02
*Smicridea*						
(*Rhyacophylax*)	1	3.89	6.74	1.25	8.22	58.24
*Atopsyche*	1.9	1.73	5.12	1.41	6.24	64.49
*Helicopsyche*	1.63	0.93	4.32	1.01	5.27	69.76
*Rhyacopsyche*	1.14	0	3.61	2.23	4.4	74.16
*Chimarra*						
(*Curgia*)	0	1.04	3.05	1.29	3.72	77.88
*Smicridea*						
(*Smicridea*)	0	1.4	2.57	0.95	3.14	81.02
*Chimarrhodella*	0	1.6	2.15	0.55	2.83	83.65
*Mortoniella*	0	0.71	1.69	0.8	2.07	85.72
*Polycentropus*	0.33	0.43	1.59	0.77	1.94	87.66
*Phylloicus*	0.33	0.5	1.4	0.84	1.17	89.36
*Amphoropsyche*	0.47	0	1.27	0.82	1.55	90.91

Average minimum air temperature (T° min) is the main variable explaining the resulting ordination (Fig 2b). The first group includes all high elevation localities above 2,000 m, characterized by harsh environmental conditions expressed in lower minimum air temperatures, a decrease in annual rainfall, coupled with smaller basins. The second group contains habitats of milder environmental conditions with higher minimum air temperatures, higher precipitation, and larger watersheds (Fig 2b, S2 Table).

### Trichoptera diversity patterns across the elevation gradient

Most of the species were recorded at a single locality, reflecting a high degree of rareness. Of the 69 species found across the elevation gradient, 53 species (77%) occurred at only a single locality, and at least 10 of those species are endemic to the western ridge of the Ecuadorian Andes, an area strongly influenced by the Chocó bioregion in terms of species composition (S2 Table).

**Fig 3:**
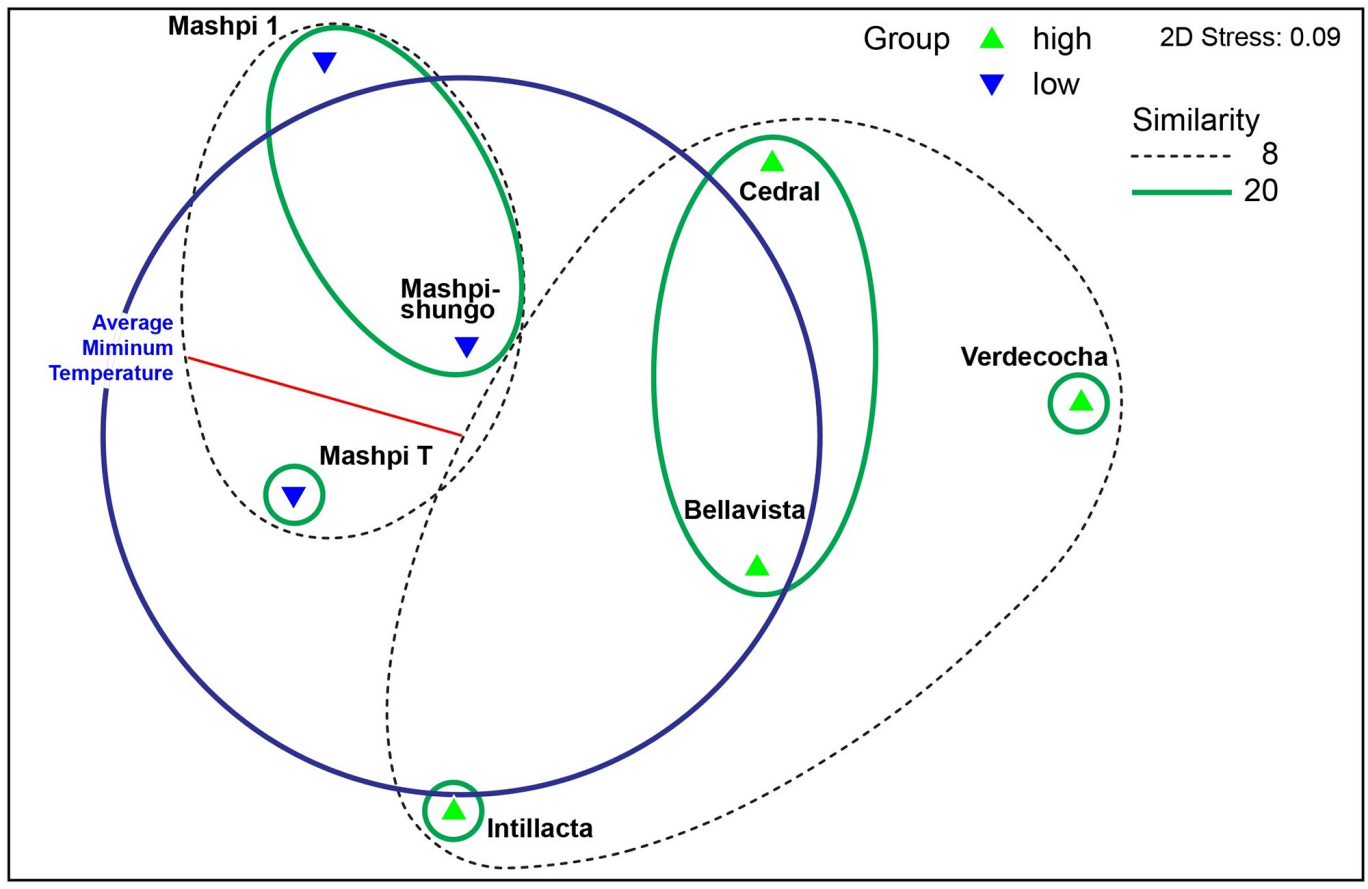
Non-metric multidimensional scaling of Trichoptera species with correlations to environmental variables (> 0.65) across an elevation gradient (600–3,500 m a.s.l.) in western Ecuador.

The ordination analysis segregated the study localities into two clearly distinguishable groups along the elevation gradient (second ordination axis)—the low elevation localities located on the left-hand side of the diagram and the mid to high elevation localities, placed at the center and on the right-hand side, where T° min was the main variable related to this configuration (Fig 3). However, the resulting groups showed very low similarity (8%), suggesting high species turnover among localities (see below).

Since we observed that locality Mashpi T had a very high richness of species and genera compared with the other localities, we repeated all the ordination and cluster analyses without this outlier, and results were similar to those observed with all localities (S1 Fig).

### Geographic distance, elevation and community composition

The matrix correlation analysis between community genus level composition and geographic distance showed a strong correlation (Rho = 0.624; p = 0.016). However, the community composition similarity at the species level was not significantly correlated with geographic distance (Rho = 0.321; p = 0.2874).

The overall community turnover between Trichoptera assemblages across the elevation gradient, independent of geographic distance, was higher for species compared to genera (Whittaker’s indices: 4.03 and 1.89, respectively; S2 and S3 Tables). Along the elevation gradient, β‐diversity of genera and species had a bimodal distribution. Genera β‐diversity showed a higher turnover at the lower section of the elevation gradient, between 903 and 1,028 m a.s.l., and a second important shift between 1,874 and 2,281 m a.s.l (Table 4). Species β‐diversity showed a bimodal distribution as well, but stronger and at different elevation ranges. The first high turnover was observed between 1,028 and 1,874 m a.s.l., and the second one between 2,325 and 3,464 m a.s.l (Table 4). The observed changes in community composition along the elevation gradient were not fully supported by the null models, neither at the species nor the generic level. The SES statistics suggest a non-conclusive random distribution of the regional species pool. However, null models strongly support the observed species turnover for localities grouped by elevation (Table 5), but not for genera. The strongest species turnover were at 1,028 to 1,874 and from 2,281 to 3,464 m a.s.l. This change in species composition is in line with the patterns observed when the entire gradient was analyzed. Although not significant, the strongest turnover for genera among elevation groups was observed at 2,281 to 3,464 m a.s.l.

**Table 4:** Species (A) and generic (B) β-diversity dissimilarity (Whitaker), mean null models (1000 interactions), and the SES score along the elevation gradient. Bold numbers are significant values according to SES.

Elevation range (m asl)	Observed	Null	SES
(A)			
619–903	0.55	0.82	-2.62
903–1028	0.76	0.81	-0.56
1028–1874	**0.87**	0.82	0.58
1874–2281	0.83	0.81	0.2
2281–2325	0.62	0.81	-1.91
2325–3464	**0.88**	0.81	0.64
(B)			
619–903	0.33	0.67	-2.35
903–1028	**0.63**	0.66	-0.27
1028–1874	0.42	0.67	-1.79
1874–2281	**0.5**	0.66	-1.14
2281–2325	0.23	0.67	-3.051
2325–3464	0.4	0.67	-2.01

**Table 5:** Species and generic β-diversity dissimilarity (Whitaker), mean null models (1000 interactions), and the SES score along sites grouped by elevation.

Elevational Groups	Elevation range (m asl)	Observed	Null	SES
	**Species**			
Low-elevation	619–1028	**0.855**	0.63	**2.78**
Intillacta (mid-elevation)	1029–1874	**0.881**	0.628	**3.185**
High-elevation	2281–3464	**0.882**	0.633	**3.181**
	**Genera**			
Low-elevation	619–1028	0.455	0.468	-0.13
Intillacta (mid-elevation)	1029–1874	0.55	0.472	0.87
High-elevation	2281–3464	**0.58**	0.466	1.06

## Discussion

Trichoptera communities showed a higher species and generic diversity at lower localities (500 to 1,000 m a.s.l.) and that then decreased with increasing elevation, confirming our first hypothesis. Although our data are constrained by low sampling effort, the observed patterns are in agreement with the occurrence data for the caddisflies of Ecuador [18]. Further, similar patterns have been documented along elevational gradients in the tropical Andes for other taxa such as rodents [42], bats [8], ferns and lycopods [5, 43], epiphytes [44], and trees [45–47].

Our data suggest that the decline in Trichoptera richness at high elevations is related to low air temperature (Fig 2a & 2b). Cold temperatures are associated to longer life cycles, that can affect diversity [45]. Another possibility is that with low air temperatures adults are less likely to fly to black lights. However, studies with sticky and Malaise emergence traps, along different seasons have found the same low richness and abundance in high altitude tropical streams [48, 49]. Localities up to 1,800 m a.s.l. have higher minimum temperatures (20.25°C—14°C) than the upper localities (7.3–3.7°C). The most abundant genera at the lowland localities (Table 3) are mainly collector-gatherers (*Chimarra*, *Wormaldia*, *Leptonema*, and *Smicridea*). The communities at localities above 2,200 m a.s.l. showed lower species and generic richness but higher functional feeding diversity (less functional redundancy). *Atopsyche* is mainly a predator, *Rhyacopsyche* and *Helicopsyche* are primarily scrapers, and *Nectopsyche* is a shredder [50, 51]. This also suggests high functional feeding diversity agrees with resource changes along rivers at different altitudes [52]. Also, this suggests that the harsher climatic conditions of the high Andes might be acting as a strong environmental filter for low elevation taxa restricted to below 2,000 m a.s.l. [13]. Recent studies in similar ecosystems and with similar taxa [53, 54] suggest that thermal conditions from higher locations seem to act as a physiological barrier, selecting for organisms with narrow physiological tolerances to temperature and limited distribution along tropical elevation gradients. Therefore, physiology and resource availability play a key role in shaping diversity gradients in Andean freshwater taxa.

Our results suggest an increase in generic caddisfly β‐diversity (taxa turnover among locations) as the geographic distance increased along the elevation gradient (RELATE procedure results). We found no significant changes at the species level, albeit a similar trend, increased community composition dissimilarity by increased geographic distance, was observed (Fig 3). These findings partially confirm our second hypothesis, although our results are constrained by low sample effort and few locations.

As expected, changes in community assemblages at the species level were higher than in generic composition. β-diversity patterns along elevational groups of localities were strongly supported by our null models, suggesting a strong, non-random, environmental filtering of Trichoptera communities (Table 5). This is in agreement with what we know of the Trichoptera fauna of Ecuador [18], in which, of the 266 species with elevation records, only 9 species are present along the whole gradient, with 47 above, and 210 below 2000 m asl. The substantial community turnover along the elevational/thermal gradient has been previously reported for other Andean aquatic insect taxa [13, 53, 54]. Complementing this observation, a recent study of aquatic insect communities in Brazil and Finland found that β‐diversity patterns of tropical communities (i.e., closer to stochastic expectations) are explained by the low number of individuals within these communities, which in turn are affected by ecological drift [55]. These authors suggested that ecological drift may drive variation in some small communities by changing the expected outcome of niche selection, increasing the chances of having communities characterized by species with low abundances and narrow distributions, which was also observed in our study.

Patterns of high endemism in the caddisfly faunas of high elevations in mountain ecosystems have been observed for several genera worldwide, such as *Drusus* in Europe [56] and *Contulma* in South America [49, 57]. Besides, species turnover among caddisfly communities in our study area was high (mean value of Whittaker’s indices: 0.85 ± 0.11), but similar to the values reported for the aquatic communities studied in Brazil (β‐diversity = 0,87) [55]. Species turnover (β‐diversity) for aquatic insects in tropical mountains can be even higher in glaciated streams suggesting that community composition dissimilarity in these insect communities increases with elevation [58, 59] and along environmental gradients.

Community generic similarity was highly correlated with geographic proximity (Rho = 0.624; p = 0.016), whereas at the species level we found no correlation (Rho = 0.321; p = 0.2874), partially confirming our second hypothesis. This high species turnover pattern suggests that for caddisfly species assemblages, environmental filters (i.e., minimum temperature) along wide elevation gradients are more important than linear geographic proximity [60]. In tropical mountain ecosystems, changes in environmental conditions over short distances become more relevant than localities closer to each other but with similar environments [21]. Thus, the bimodal pattern observed in the species β‐diversity confirms the occurrence of sharp temperature shifts at 1,874 and 3,464 m a.s.l (Table 5). Lastly, shifts in community composition were dominated by the loss of species, rather than their gain, at higher elevations.

### Conservation implications

The high Trichoptera β‐diversity found in this elevation gradient suggests that communities are highly ecologically specialized, with narrow range species and low abundances [18, 57]. Climate change is forcing species to move upwards in mountain environments [61]. We expect that tropical lowland Trichoptera that show narrower thermal tolerances compared to mid and high elevation species [13, 54], face greater risk of extinction for lowland than highland species in the face of rising temperatures. Coupled with climate change, habitat destruction, overexploitation, pollution, and reduction in connectivity are the primary drivers of freshwater ecosystem degradation [55, 62]. For mid and high-elevation taxa, the habitat degradation that leads to loss of functional connectivity is a major threat [63]. The fact that geographic distance and elevation explained a high percentage of community variance across the studied elevation gradient and the aforementioned threats, highlights the need for conserving river networks along the elevation gradient to effectively protect Trichoptera diversity.

## Supporting information

S1 Fig Natural vegetation cover across the elevation gradient. (TIF)

S2 Fig Species accumulation curves for species collected and CHAO1 species estimators for each of the localities sampled. (TIF)

S3 Fig Sample coverage across localities. (TIF)

S1 Table Complete dataset that contains species collected per site, date with abundances and sex ratios. (XLSX)

S2 Table Dissimilarity (pairwise comparison) of Trichoptera genera community composition between localities. (DOCX)

S3 Table Dissimilarity (pairwise comparison) of Trichoptera species community composition between sampling localities. (DOCX)
