# Supplementary figures and images for: Elevational biodiversity gradients in the Neotropics: Perspectives from freshwater caddisflies (Insecta: Trichoptera)
Source: PLoS One. 2022 Aug 5;17(8):e0272229. doi: 10.1371/journal.pone.0272229 (PMC9355261; doi:10.1371/journal.pone.0272229)

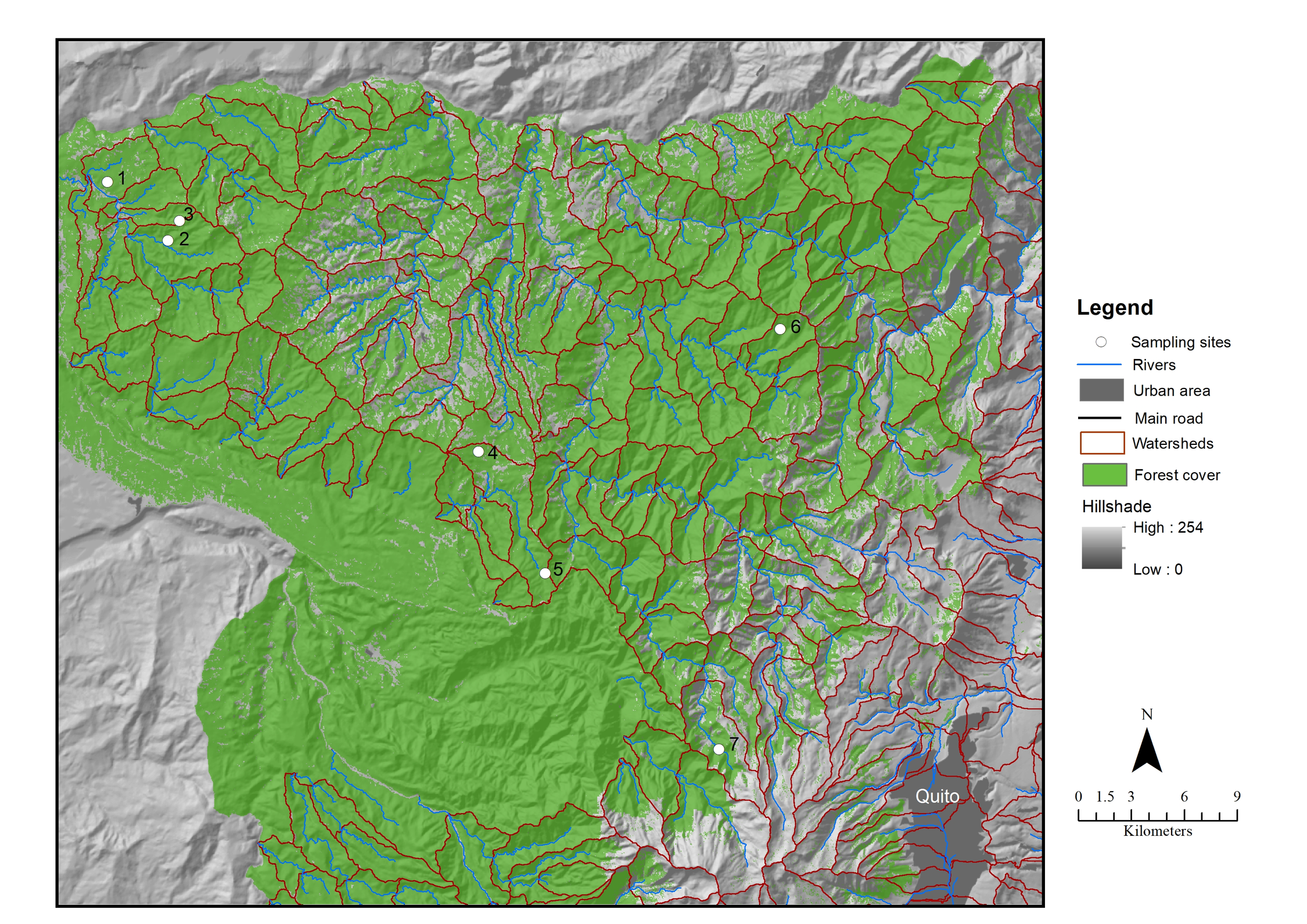

Supplement: S1 Fig — (TIF) [file pone.0272229.s001.tif]

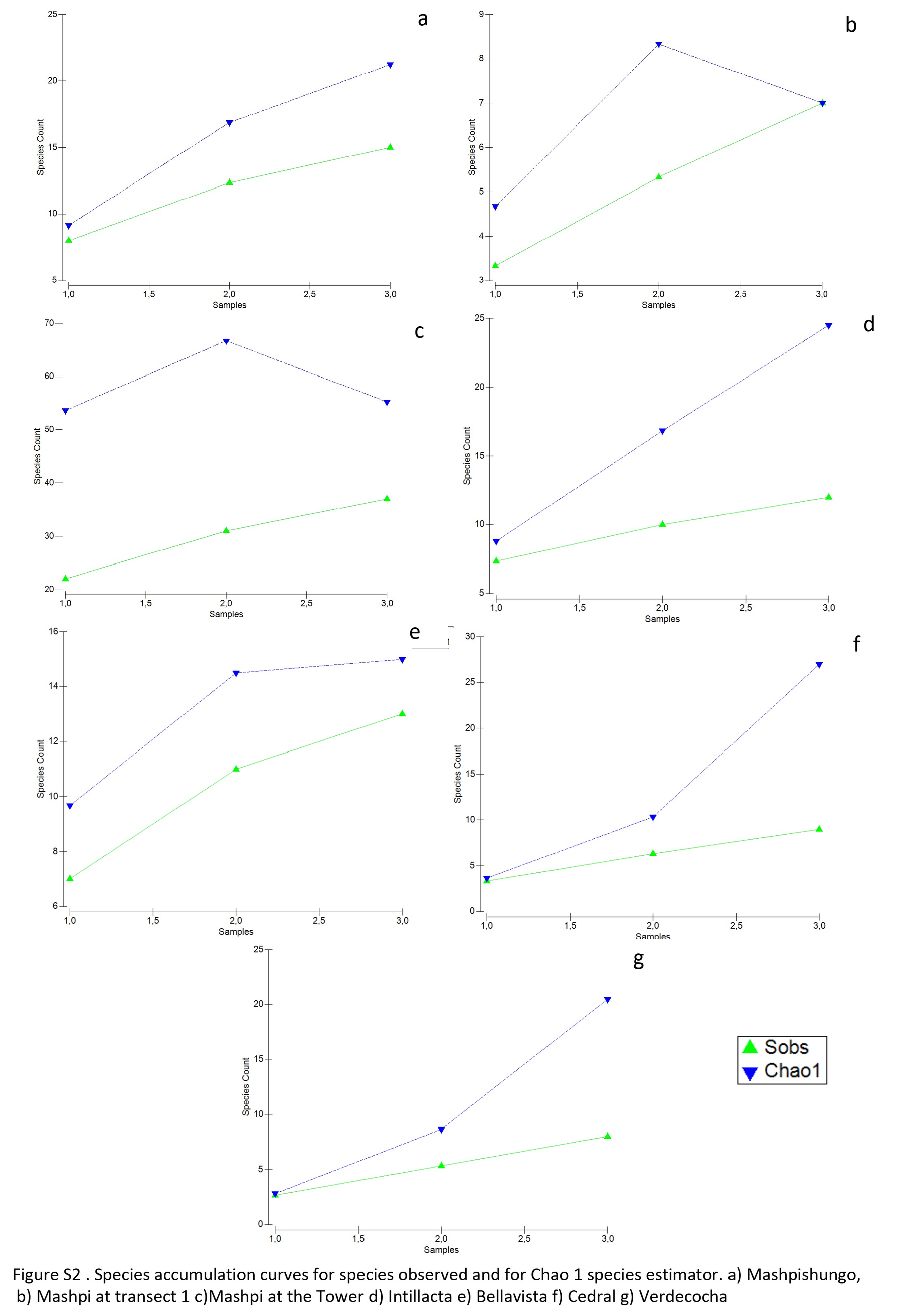

Supplement: S2 Fig — (TIF) [file pone.0272229.s002.tif]

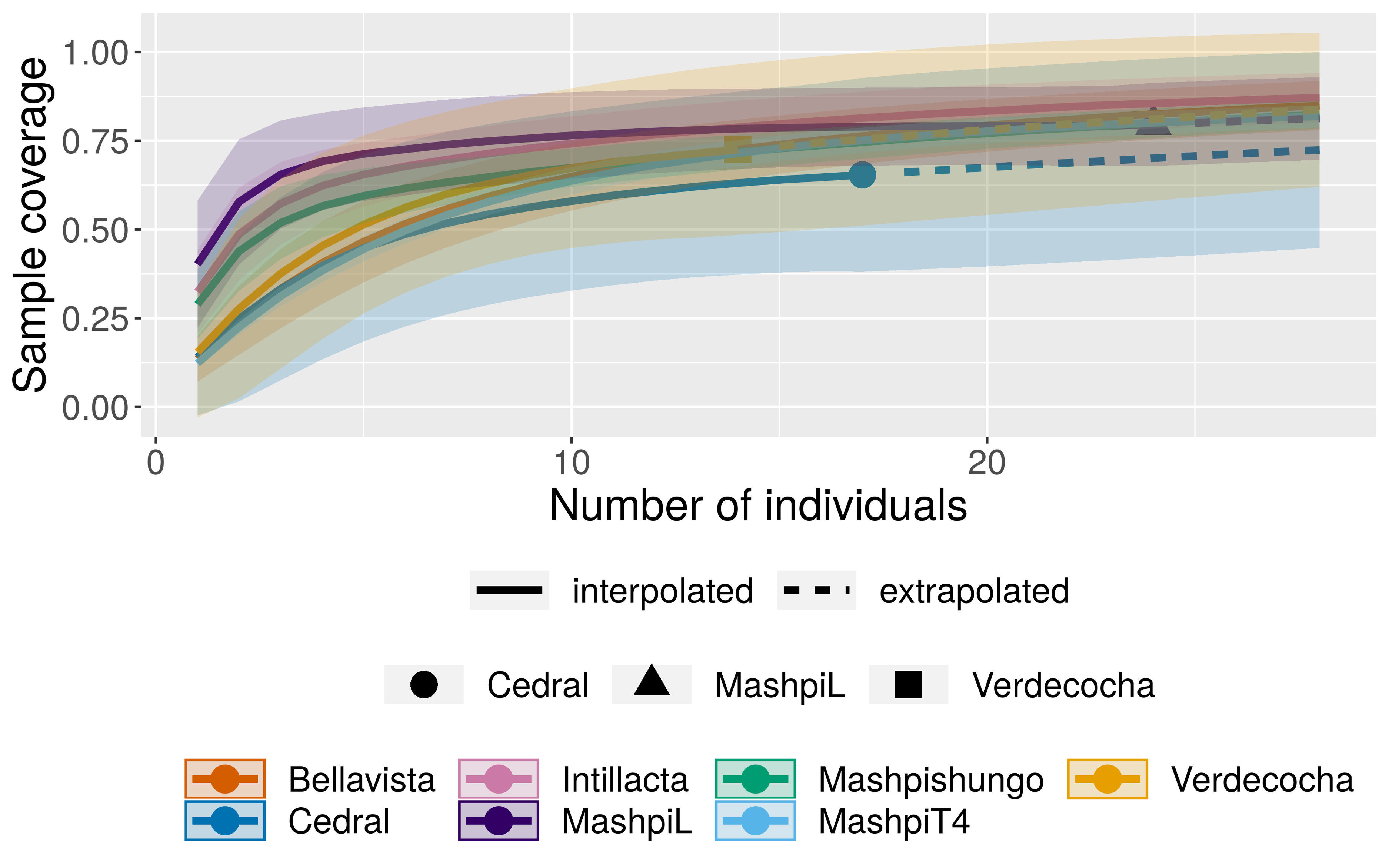

Supplement: S3 Fig — (TIF) [file pone.0272229.s003.tif]
