# Supplementary material for: Elevational biodiversity gradients in the Neotropics: Perspectives from freshwater caddisflies (Insecta: Trichoptera)
Source: PLoS One. 2022 Aug 5;17(8):e0272229. doi: 10.1371/journal.pone.0272229 (PMC9355261; doi:10.1371/journal.pone.0272229)
Supplement: S3 Table — (DOCX) [file pone.0272229.s006.docx]

S3 Table. Dissimilarity (pairwise comparison) of Trichoptera species community composition between sampling locations.

|  | Verdecocha | Cedral | Bellavista | Intillacta | Mashpi T | Mashpi 1 | Mashpishungo |
| --- | --- | --- | --- | --- | --- | --- | --- |
| Verdechocha | 0 | 0.88 | 0.89 | 0.89 | 0.95 | 1 | 0.91 |
| Cedral |  | 0 | 0.62 | 0.9 | 0.86 | 0.88 | 0.83 |
| Bellavista |  |  | 0 | 0.83 | 0.87 | 1 | 0.93 |
| Intillacta |  |  |  | 0 | 0.87 | 0.89 | 0.85 |
| Mashpi T |  |  |  |  | 0 | 0.76 | 0.72 |
| Mashpi 1 |  |  |  |  |  | 0 | 0.55 |
| Mashpishungo |  |  |  |  |  |  | 0 |
